# Local Microenvironment Provides Important Cues for Cell Differentiation in Lingual Epithelia

**DOI:** 10.1371/journal.pone.0035362

**Published:** 2012-04-13

**Authors:** Feng Li, Mingliang Zhou

**Affiliations:** 1 Laboratory of Developmental Biology, Xinhua Hospital, School of Medicine, Shanghai Jiao Tong University, Shanghai, People's Republic of China; 2 Monell Chemical Senses Center, Philadelphia, Pennsylvania, United States of America; 3 Department of Laboratory Animal Science, School of Medicine, Shanghai Jiao Tong University, Shanghai, People's Republic of China; Cincinnati Children's Hospital Medical Center, United States of America

## Abstract

Transgenic Keratin14-rtTA-PTR mice specifically express Keratin14 (K14) in the tongue epithelia, as well as co-express *EGFP* and the dominant negative *ΔTgfbr2* genes upon treatment with Doxycycline (Dox). As TGF-β signaling negatively regulates the stem cell cycle and proliferation, its disruption by Dox induction in these transgenic mice shortens the cell cycle and allows observation of the final fate of those mutated cell lineages within a short period of time. Here, we used inducible transgenic mice to track the K14+ cells through the cell migration stream by immunohistochemical an immunofluorescent imaging. We showed that these cells have different development patterns from the tip to posterior of the tongue, achieved presumably by integrating positional information from the microenvironment. The expression of the *K14* gene was variable, depending on the location of the tongue and papillae. Disruption of TGF-β signaling in K14+ progenitor cells resulted in proliferation of stem cell pools.

## Introduction

The specialized mucosa of the mouse tongue contains numerous papillae of three main types, filiform, fungiform and circumvallate (CV). A fourth type located at the edges of the tongue is the foliate papillae. Taste buds (TB) are present on the fungiform, CV and foliate papillae, the most numerous of which are the filiform papillae [1]. However, the mechanism of papillae formation is still presently unclear. In a previous study [2], the pattern of labeled basal cells was demonstrated to vary 45 min after injection of H3T, depending on the area of the tongue, and the calculated turnover time of cells in the basal layer was different, depending on the location in the tongue (dorsal surface: tip of tongue, 32 h; middle of tongue, 40 h; back of tongue, 53 h; ventral surface: 46 h). That study not only showed that the tongue epithelium is one of the most rapidly self-renewing tissues in adult mammals, it also suggested a variable development of tongue epithelial cells based on the area of the tongue in which they are located.

The tongue has many relationships and connections in the body, both to the meridians and the internal organs according to traditional Chinese medicine. For clinical purposes, observations of the tongue, or tongue diagnosis, can provide strong visual indicators of an individual's overall harmony or disharmony, although the molecular mechanisms to support tongue diagnosis have yet to be determined. In addition, how epithelia are formed and maintained is one of the key problems of developmental biology and an area in which many basic questions remain unresolved. For example, cell specialization was originally thought to be simply a reflection of differential gene expression, and the fate of a stem cell population is pre-determined by internal regulatory processes [3], [4], [5], [6]. Microenviromental cues can re-direct epithelial cell fate, allowing lateral movement and crossing of primitive germ layer boundaries [7].

It has been shown that multiple stem cell populations exist in the lingual epithelia, including Keratin14+ (K14) progenitor cells [8], [9]. After crossing with a transgenic mouse line carrying an EGFP-pBi-DeltaTgfbr2 construct (PTR) [10], animals expressing rtTA under the control of the K14 promoter will show cell-type-specific expression of a dominant-negative TGF-β type II receptor. Many studies have revealed that TGF-β signaling plays an important role in growth inhibition and arresting cell cycle [11], [12], [13], [14], [15]. Provided that absence of TGF-β signaling shortens the cell cycle without affecting the fate of mutant cells [14], this model allowed us to track the fate of K14+ progenitor cells and to preliminarily investigate the molecular mechanisms affecting spatial development of these cells in the adult tongue after disruption of TGF-β signaling *in vivo*. Meanwhile, the formation mechanism of filiform papillae was also investigated by immunohistochemical and immunofluorescent imaging. As the current understanding of epithelial stem cells is strongly influenced by *in vitro* studies [16], [17], [18], [19], these results will help us to understand the role of the microenviroment during the development of epithelial stem cells *in vivo*, which is a key step towards uncovering the molecular basis of tongue diagnosis.

## Results

### Differential developmental speed of epithelial cells from tip to posterior of the tongue and variable K14 gene expression are dependent on the location on the tongue and papillae

In a previous cell genesis model of the tongue, multiple populations of stem cells have been identified, including K14+ and Sox2+ progenitor cells [8], [9]. K14+ cells continuously give rise to both mature TB receptor cells and surrounding keratinocytes [8]. In order to further investigate the nature of K14+ progenitor cells, we crossed K14-rtTA (X-linked K14-rtTA transgene) mice with PTR mice [20]. The double-transgenic K14-rtTA-PTR mice specifically express K14 in the lingual epithelia and also co-express *EGFP* and the dominant negative *ΔTgfbr2* genes upon treatment with Doxycycline (Dox) [10].

Adult K14-rtTA-PTR mice were exposed to Dox and sacrificed at 5 h, 9 h, 1, 3, 7 and 35 days after induction. As the rtTA protein is kept in daughter cells originating from K14 progenitors due to a shortened cell cycle after disruption of TGF-β signaling [14], Dox induction should continually induce GFP expression in those daughter cells (Figure S1). With extended exposure to Dox, GFP+ cells should gradually be found in the tongue epithelia and papillae.

**Figure 1:**
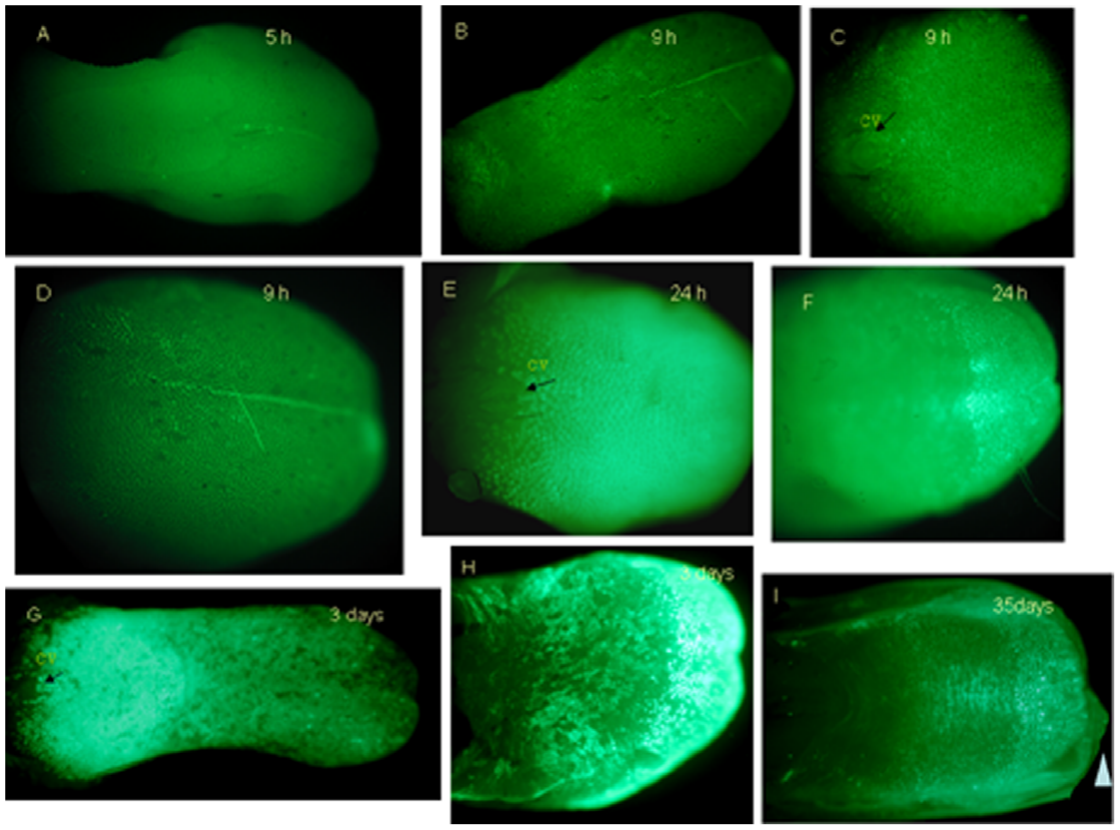
GFP expression in tongue over time after Dox induction in K14-rtTA-PTR mice. In K14-rtTA-PTR mice, no GFP expression was observed after 5 h of Dox induction (A). However, GFP appeared in the posterior of the tongue after 9 h of Dox induction (B and C), and few GFP+ cells appear in the anterior one third (D). After 1 day of Dox induction, obvious GFP expression was found on the dorsal surface (E) and ventral surface (F). After 3 days of Dox induction, GFP expression was distributed on the whole dorsal surface of the tongue (G). GFP expression was also observed in both papillae and non-papillae areas of the ventral surface (H). After 35 days of Dox induction, an abnormal tip of the tongue was found (I, triangle). Arrow indicates CV papillae (C, E, F).

In K14-rtTA-PTR mice, we failed to observe GFP expression after 5 h of Dox induction (Figure 1A). However, GFP appeared in the posterior (Figure 1B and 1C) and middle (Figure 1B and 1D) of the tongue after 9 h of Dox induction. After 1 day of Dox induction, obvious GFP expression was found on the tongue surface, including the dorsal surface (Figure 1E) and ventral surface (Figure 1F). After 3 days of Dox induction, GFP expression was obviously distributed throughout the dorsal surface of the tongue (Figure 1G). GFP expression was also observed in both papillae and non-papillae areas of the ventral surface (Figure 1H). After 35 days of Dox administration, an abnormal tip of the tongue was found (Figure 1I, triangle).

**Figure 2:**
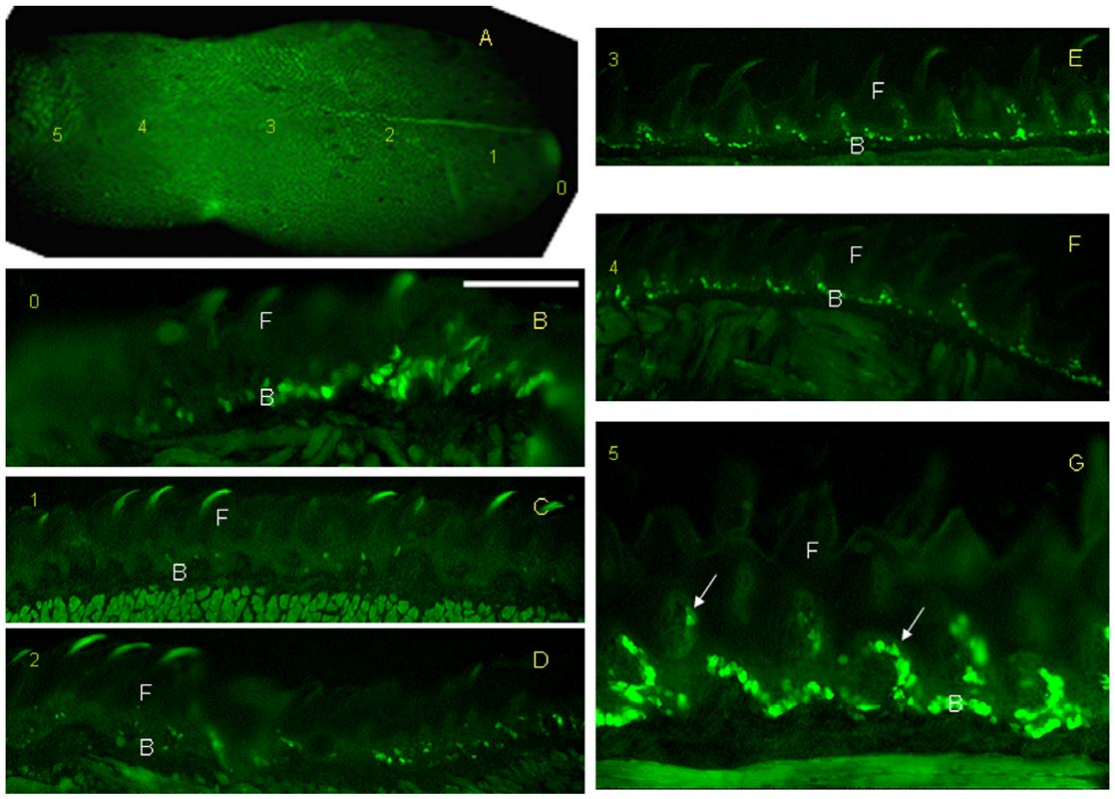
GFP expressions are differentially distributed along the tongue epithelia after 9 h of Dox induction in K14-rtTA-PTR mice. The tongue was sectioned into six parts from tip to posterior (sites 0–5, A). After 9 h of Dox induction, the frontal section showed some GFP+ cells in the basal cell layer at site 0. Those GFP+ cells were concentrated on the dorsal surface near the middle line of the tongue (B). Sagittal sectioning showed few GFP+ cells at site 1. A few GFP+ cells appeared at site 2 (D). Some GFP+ cells appeared in the basal cell layer at sites 3 and 4 (E, F). Some GFP+ cells were also observed in the basal cell layer at site 5 with frontal sectioning. Noted that GFP+ cells appeared on the interior of the papillae (G arrow). F filiform papillae. B, basal cell. Scale bar, 80 µm.

Furthermore, a gradual pattern of GFP+ cell expression was observed by serial sagittal sectioning of the tongue from K14-rtTA-PTR mice treated with Dox. The tongue was separated into six parts from tip to posterior, and indicated by site numbers from 0–5 (Figure 2A). After 9 h of Dox induction, the frontal section showed some GFP+ cells in the basal cell layer at site 0 (tip). Those GFP+ cells were concentrated on the dorsal surface near the middle line of the tongue (Figure 2B). Sagittal sectioning showed a few GFP+ cells at site 1 (Figure 2C) and site 2 (Figure 2D). More GFP+ cells appeared in the basal cell layer at sites 3 and 4 (Figure 2E and 2F) than at sites 1 and 2, and some GFP+ cells were also observed in the basal cell layer at site 5 in the frontal section (Figure 2G). Interestingly, we did not find GFP expression on the ventral surface along the entire length of the tongue (data not shown). We also noted that the GFP+ cells all appeared in the basal cell layer, indicating that the fate of the K14+ cells was to develop into basal cells.

**Figure 3:**
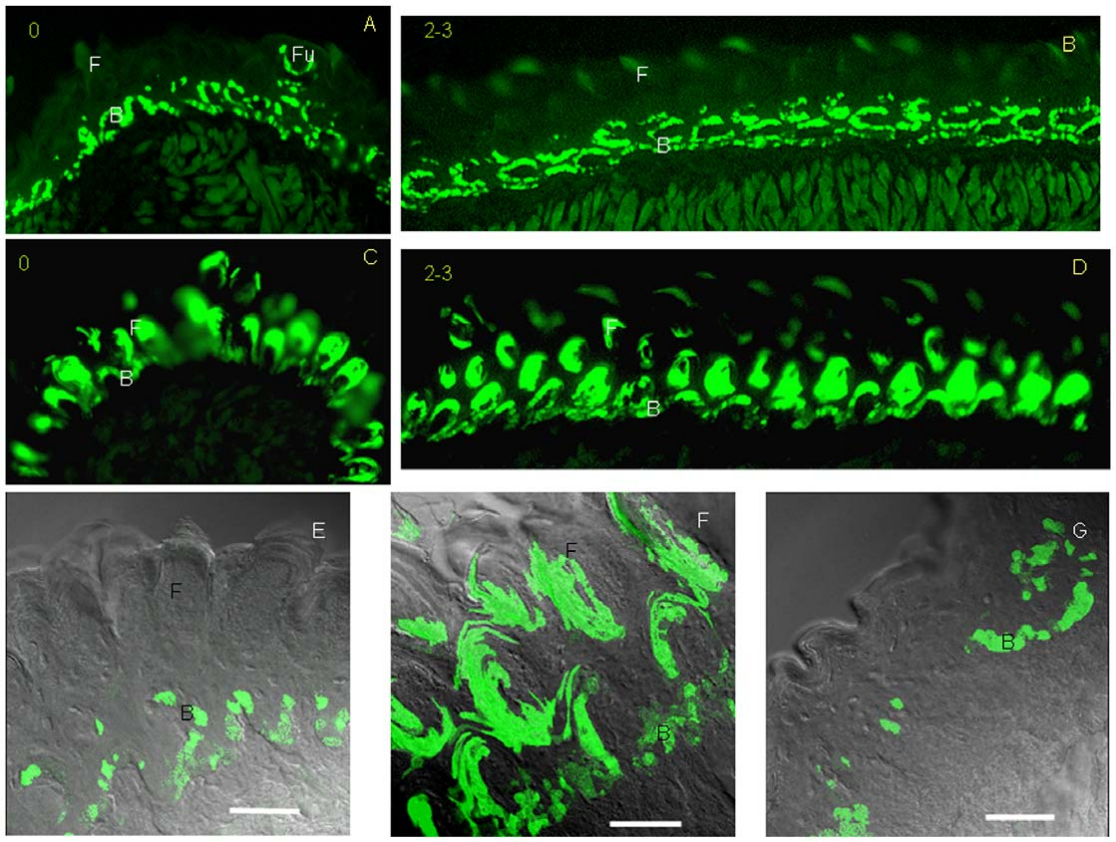
GFP expression was observed in filiform papillae after 3 days of Dox induction in K14-rtTA-PTR mice. After 24 h of Dox induction, GFP+ cells appeared in the basal cell layer (A, B). After 3 days of Dox induction, GFP+ cells appeared in the filiform papillae and basal cell layer (C, D). GFP+ cells appeared in the basal cell layer after 24 h of Dox induction (E, high magnification). After 3 days of Dox induction, GFP+ cells appeared at the filiform papillae and basal cell layer on the dorsal surface (F, high magnification). GFP+ cells were only observed in the basal cell layer (G, high magnification). F filiform papillae. Fu, fungiform papillae. B, basal cell. 0: site 0; 2–3: sites 2–3. A, C, frontal section; B, D: sagittal section. Scale bar, 50 µm for E F and G.

After 24 h of Dox induction, GFP+ cells appeared in the basal cell layer along the whole lingual epithelia (Figure 3A and 3B). After 3 days of Dox induction, GFP+ cells appeared in the filiform papillae and basal cell layer (Figure 3C and 3D). Confocal analysis further showed that GFP+ cells first appeared in the basal cell layer after 24 h of Dox induction (Figure 3E). After 3 days of Dox induction, GFP+ cells appeared in the filiform papillae and basal cell layer on the dorsal surface (Figure 3F). However, GFP+ cells were only observed in the basal cell layer in non-papillae epithelia.

Immunohischemical analysis further detected GFP expression in the basal cell layer, base of filiform papillae and TB of fungiform papillae after 24 h of TGF-β signaling disruption (Figure S2A). After 7 days of TGF-β signaling disruption, GFP expression was detected in the basal cell layer and base of filiform papillae. Furthermore, GFP expression was also detected in the spine of filiform papillae and epithelia of fungiform papillae (Figure S2B).

**Figure 4:**
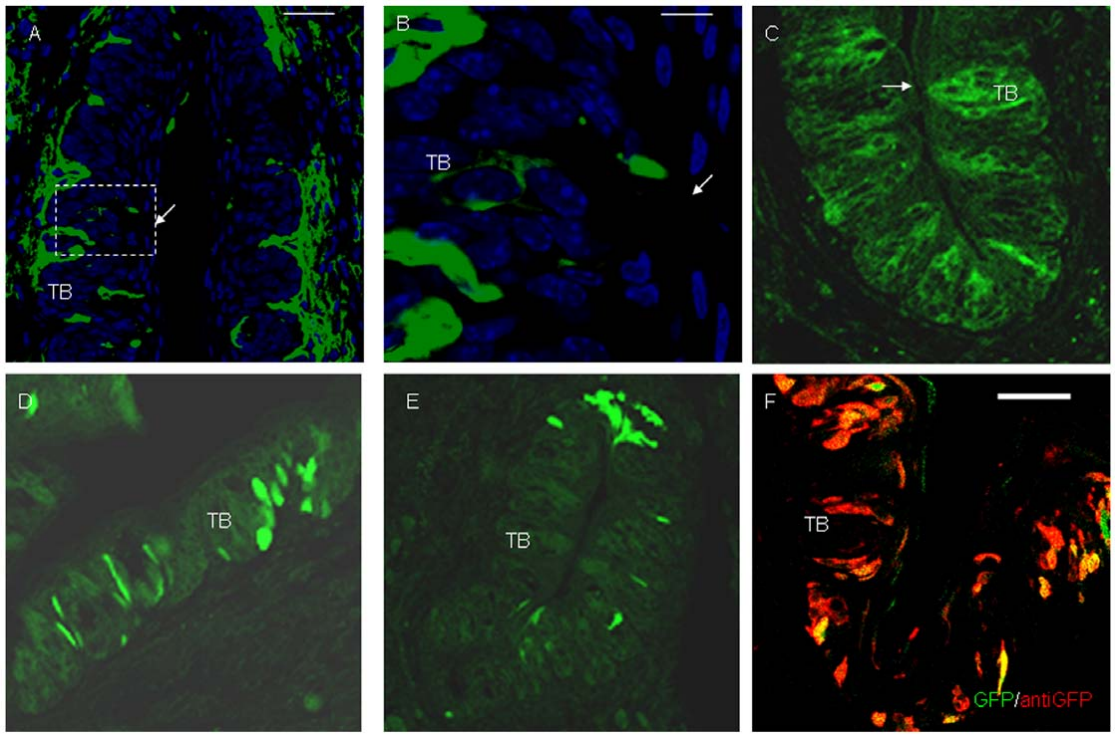
GFP expression in TB after Dox induction in K14-rtTA-PTR mice. In control mice, K14 expression was detected in TB. (A) K14 expression in CV papillae and connective tissue. (B) High magnification of TB (from dotted frame in A). After 5 h of Dox induction, weak GFP expression was found in TB of CV papillae (C). After 1 day of Dox induction, stronger GFP expression was observed in TB and epithelia around CV papillae (D). GFP expression was observed in the epithelia of CV papillae after 3 days of Dox induction (E). After 7 days of Dox induction, immunostaining with anti-GFP showed that GFP+ cells were distributed in the epithelia of CV papillae (F). Arrow, taste pore. Scale bars, 50 µm for A, C, D, E and F, and 10 µm for B.

K14 expression was detected in the TB of CV papillae and connective tissue in control mice (Figure 4A and 4B). After 5 h of Dox induction, weak GFP expression was found in the TB of CV papillae (Figure 4C). After 1 day of Dox induction, stronger GFP expression was observed in the TB and epithelia around CV papillae (Figure 4D). GFP expression was still observed in the epithelia of CV papillae after 3 days of Dox induction (Figure 4E). After 7 days of Dox induction, immunostaining with an anti-GFP antibody showed that GFP+ cells were distributed in the epithelia of CV papillae (Figure 4F).

Those results indicated a different developmental speed for lingual epithelia from tip to posterior. Less than 24 h in the TB and 9 h in lingual epithelia was needed for the K14+ progenitor cells to mature after disruption of TGF-β signaling. It should be noted that a normal maturation process from K14+ progenitor cells to mature cells is more than 3 days in lingual epithelia [8]. These results also suggested that the K14+ progenitor cells were in different stages in the model of stem cell development [3], [21], depending on the location in the tongue; that is, the further downstream the K14+ progenitor cells were located in the multiple stem cell population, the earlier GFP appeared in the basal layer. In addition, we also noted that the GFP+ cells all appeared in the basal layer, indicating that the fate of the K14+ progenitor cells was to develop into basal cells. The ectopic GFP expression in filiform and CV papillae indicated that the local microinviroment played a highly important role in regulating gene expression, and the expression of the K14 gene was variable, depending on the location of the tongue and papillae.

### Genetic tracing of K14+ lineage cells

**Figure 5:**
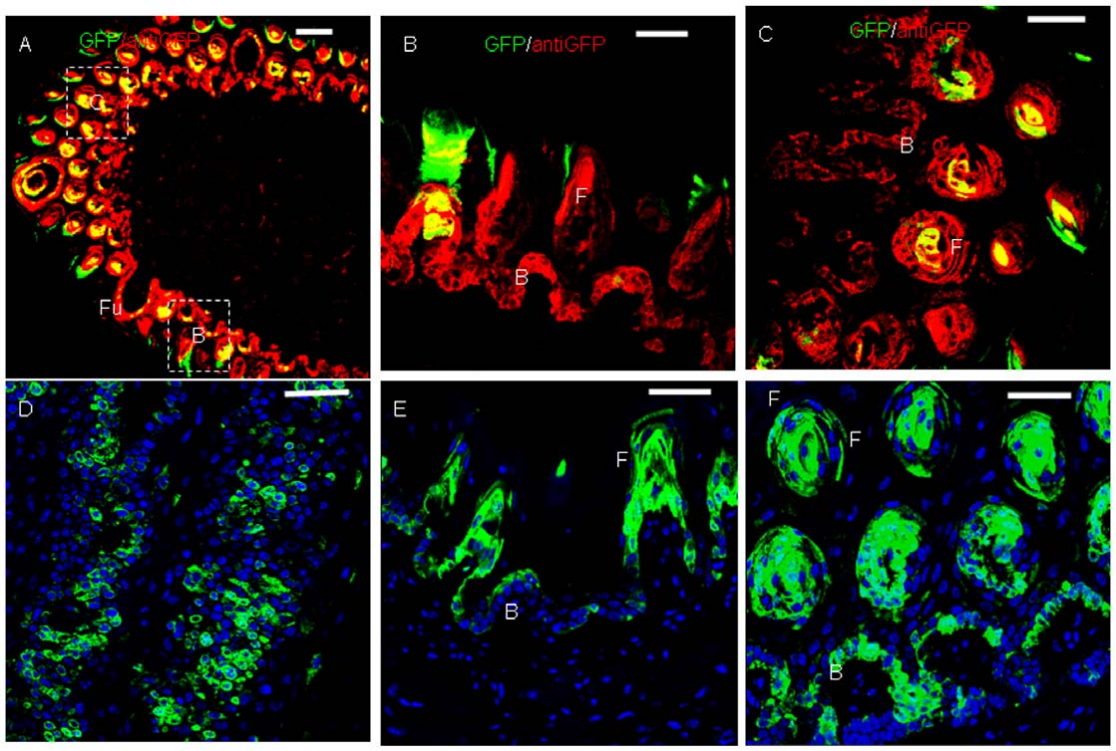
Genetic tracing of K14+ lineage cells. Confocal analysis with anti-GFP was used to trace the K14+ lineages. Immunostaining with anti-GFP showed widespread GFP expression in lingual epithelia after 7 days of Dox induction. GFP+ cells were distributed in the basal cell layer and filiform papillae. (A) Low magnification, frontal section of tip of tongue. (B) High magnification from dotted frame in B. (C) High magnification from dotted frame in C. After 35 days of Dox induction, GFP+ cells were observed in the invasion area of the ventral surface (D). GFP+ cells were observed in the basal cell layer and filiform papillae (E and F). Fu, fungiform papillae. F, filiform paillae. B, basal cell layer. TB, taste bud. Scale bar, 60 µm for A, 50 µm for B–F.

According to the model of stem cell development [3], [21] and the regulatory model of the TGF-β signaling pathway [22], [23], [24], [25], [26], [27], [28], blocking TGF-β signaling would activate and promote proliferation of K14+ progenitor cells. Therefore, GFP should gradually increase over time after Dox administration due to the distribution of the rtTA protein into daughter cells from the K14+ lineage cells. Here, confocal analysis with an anti-GFP antibody was used to trace the K14+ lineage cells. As expected, the immunostaining showed widespread GFP expression in lingual epithelia after 7 days of Dox induction. GFP+ cells were distributed in the basal cell layer and filiform papillae (Figure 5A–5C). After 35 days of Dox induction, GFP+ cells were detected in the invasion area of the ventral surface (Figure 5D). Furthermore, GFP+ cells were observed in the basal cell layer and filiform papillae including spine of filiform papillae (Figure 5E and 5F). These results collectively showed that K14+ lineage cells contributed to the development of basal cells and filiform papillae.

### Chimeric GFP expression in female K14-rtTA-PTR mice

In order to further determine the expression pattern of the *K14* gene in lingual epithelia, we analyzed the GFP expression in lingual epithelia of female mice. Since K14-rtTA is an X-linked transgene [20], disruption of the TGF-β signaling pathway in females would perhaps have less of an effect on the microenviroment, which is believed to play an important role in regulating the development/differentiation of stem cells [7], [28], [29]. Thus, the observation of GFP expression in female mice should reflect the true expression pattern of the *K14* gene.

**Figure 6:**
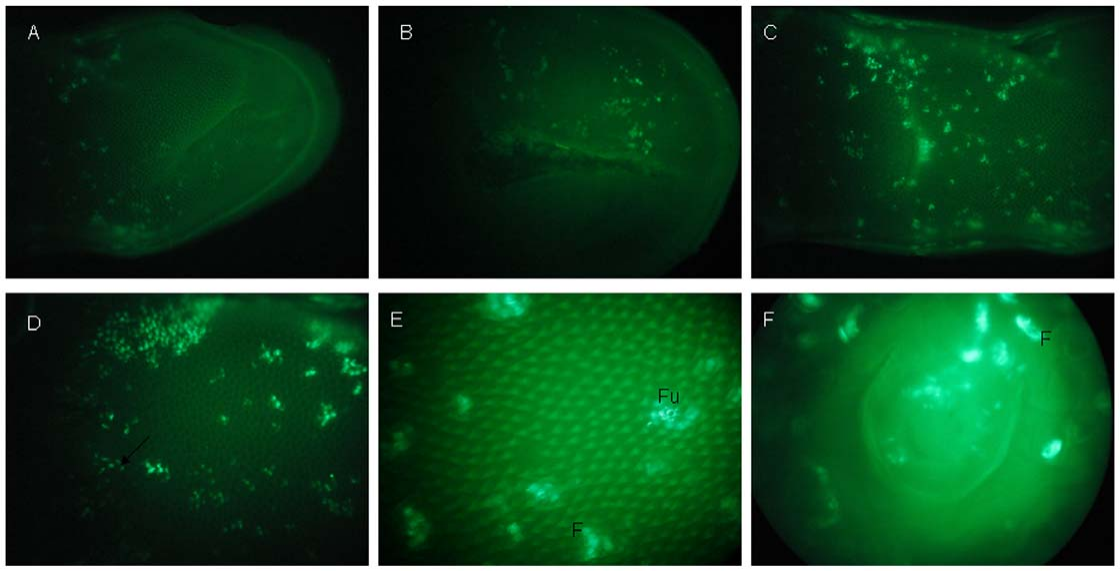
Distribution of GFP expression in the female mouse tongue after 2 days of Dox induction. Grossly, fewer GFP expressing cells were observed on the anterior one third of the dorsal surface. (A) Anterior one third of dorsal surface. (B) Ventral surface of tongue. More GFP expressing cells were observed on the posterior two thirds of dorsal surface. (C) Middle dorsal surface of tongue. (D) Posterior dorsal surface of tongue. GFP expression was observed in fungiform papillae and filiform papillae on the dorsal surface of tongue. (E) Middle dorsal surface. (F) CV papillae. Fu, fungiform papillae. F, filiform papillae. Arrow, CV papillae.

**Figure 7:**
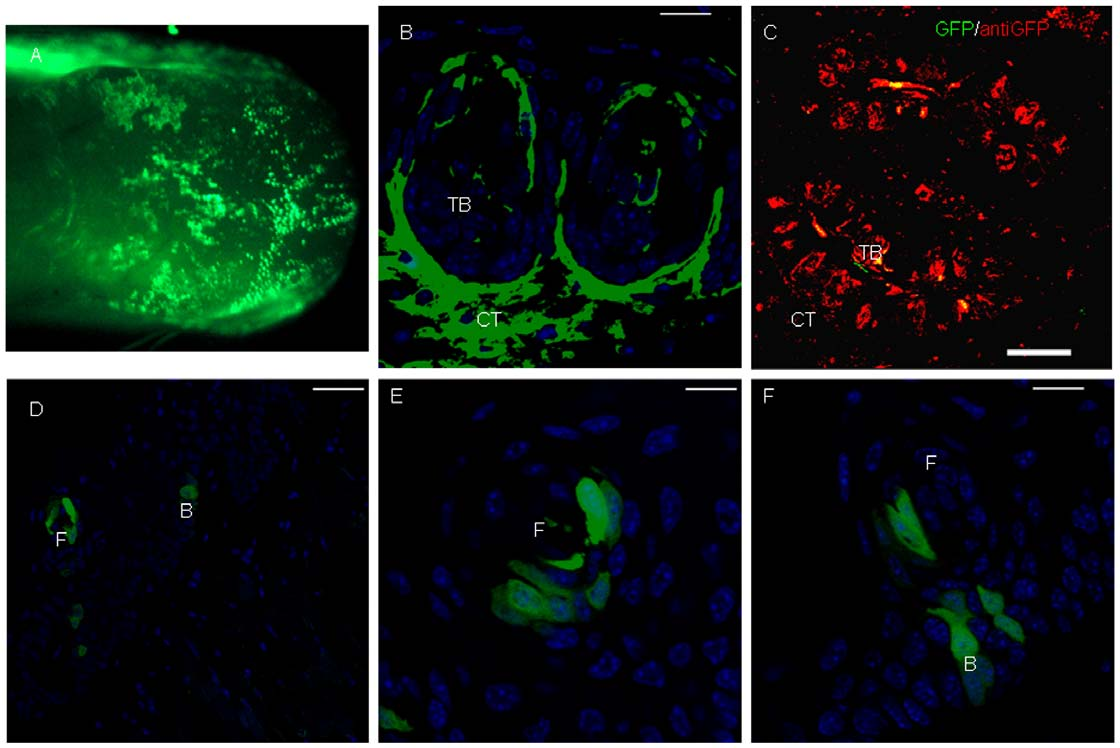
Chimeric expression of GFP in female tongue after 18 days of Dox induction. After 18 days of Dox induction in female mice, we observed chimeric expression of GFP on the tongue surface (A). Immunostaining revealed K14 expression in TB and connective tissue (B). Confocal analysis with anti-GFP further showed that K14+ lineage cells indeed contributed to TB of CV papillae (C). In lingual epithelia, GFP+ cells were mainly distributed in the basal cell layer and filiform papillae (D E and F). B, basal cell layer. CT, connective tissue. F, filiform papillae. TB, taste bud. Scale bar 10 µm for, 120 µm for C, and 50 µm for D–F.

As observed in male mice after 9 h of TGF-β signaling disruption, GFP expression was obvious in the tongue of female mice that had received 2 days of Dox induction. Grossly, fewer GFP+ cells were observed in the anterior one third of the dorsal surface (Figure 6A and 6B), while more were observed in the posterior two thirds of the dorsal surface (Figure 6C and 6D). We also observed the GFP expression in fungiform papillae and filiform papillae (Figure 6E and 6F). After 18 days of Dox induction, we still observed chimeric expression of GFP in lingual epithelia (Figure 7A). Confocal analysis showed that K14 was detected in the TB of CV papillae (Figure 7B). Furthermore, staining with anti-GFP revealed that K14+ progenitor cells indeed contributed to the TB cells of the CV papillae (Figure 7C). In lingual epithelia, GFP+ cells were mainly distributed at the basal cell layer and filiform papillae (Figure 7D–7F). Genetic tracing analysis of K14+ lineage cells with anti-GFP further showed that K14+ lineage cells were distributed at the basal cell layer, filiform papillae and spine of filiform papillae (Figure S3A and S3B). In short, the current results suggested that the K14+ progenitor cells indeed gave rise to TB cells and basal cells. Combined with results from male mice, the data revealed the distribution pattern of K14 expression along the tongue and indicated that K14+ lineage cells contributed to basal cell and filiform papillae.

### Disruption of TGF-β signaling in K14+ progenitor cells results in proliferation of stem cell pools

It was recently suggested that in the classical hierarchy of the tissue-specific stem cells, transit amplifying cells (TACs) and differentiated cells are not always rigid and irreversible [30]. That is, different epithelial stem cell populations may be functionally equivalent and interconvertible [31]. As disruption of TGF-β signaling in K14+ cells rapidly decreases the corresponding cell populations, there should be a requirement for an increase in the proportion of proliferating cells according to the epidermal proliferative unit (EPU) model [32].

**Figure 8:**
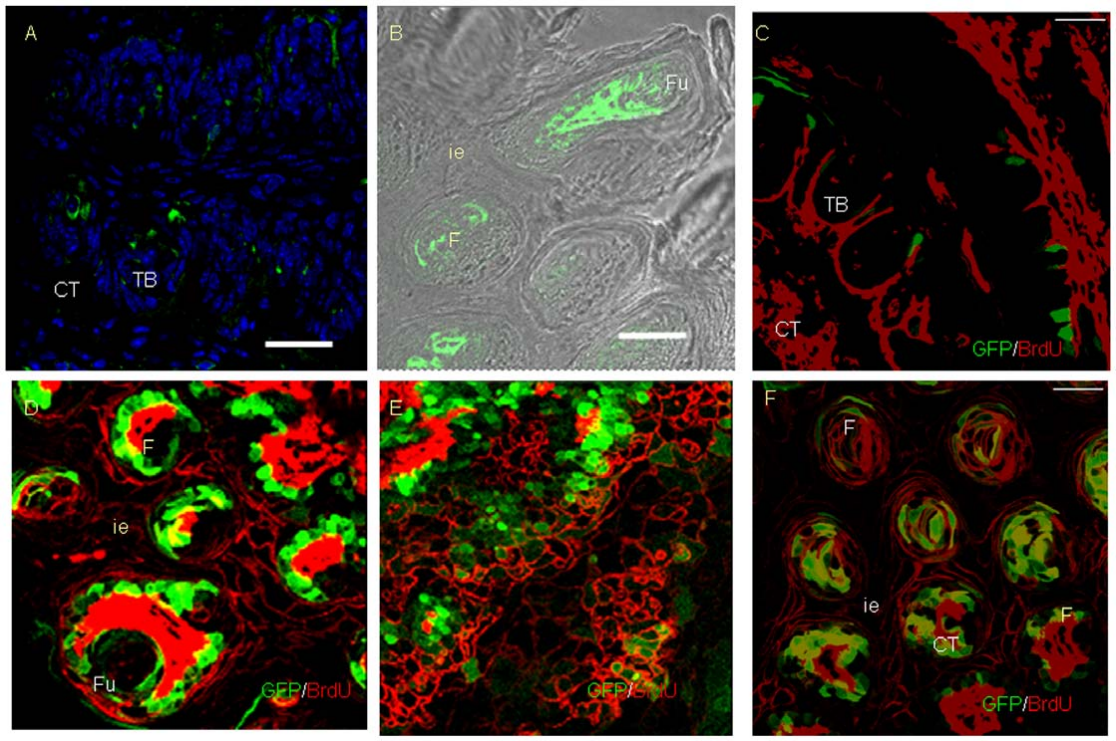
BrdU labeled proliferating cells of lingual epithelia after 35 days of Dox induction in K14-rtTA-PTR mice. In control mice, 2H BrdU injection only labeled a few cells in the CV papillae (A), fungiform and filiform papillae (B). After 35 days of Dox induction, stronger staining was shown in connective tissue around CV papillae after 2H BrdU injection (C). 2H BrdU labeled proliferating cells in lingual epithelia and connective tissue. Note that GFP partially co-localized with BrdU in lingual epithelia. In addition, 2H BrdU also revealed a cell migration stream in epithelia of the tip of the tongue. (D) Fungiform papillae. (E) Epithelia of ventral surface. Sagittal sectioning also showed 2H BrdU labeled proliferating cells in filiform papillae, connective tissue and interpapillary epithelia. Proliferating cells formed a special structure in filiform papillae (F). CT, connective tissue. F, filiform papillae. Fu, fungiform papillae. Ie, interpapillary epithelium. Scale bars, 50 µm.

In order to verify the above hypothesis, we performed a BrdU labeling experiment. In control mice, 2H BrdU injection only labeled a few cells in CV (Figure 8A), fungiform and filiform papillae and connective tissue (Figure 8B). After 35 days of Dox induction, stronger staining was shown in the connective tissue after 2H BrdU labeling (Figure 8C and 8D). Meanwhile, we also observed BrdU+ cells in the TB (Figure 8C). More interestingly, the injected 2H BrdU labeled many proliferating cells in lingual epithelia (Figure 8D–8F). On the other hand, sagittal sectioning showed that the proliferating BrdU+ cells, comprised a unique structure of filiform papillae. BrdU+ cells formed a stream in filiform papillae, which were related to that in the epithelia (Figure 8F). Immunohistochemistry further verified that the injected 2H BrdU labeled a cell migration stream that was formed by proliferating cells after 35 days of TGF-β signaling disruption (Figure S4B).

**Figure 9:**
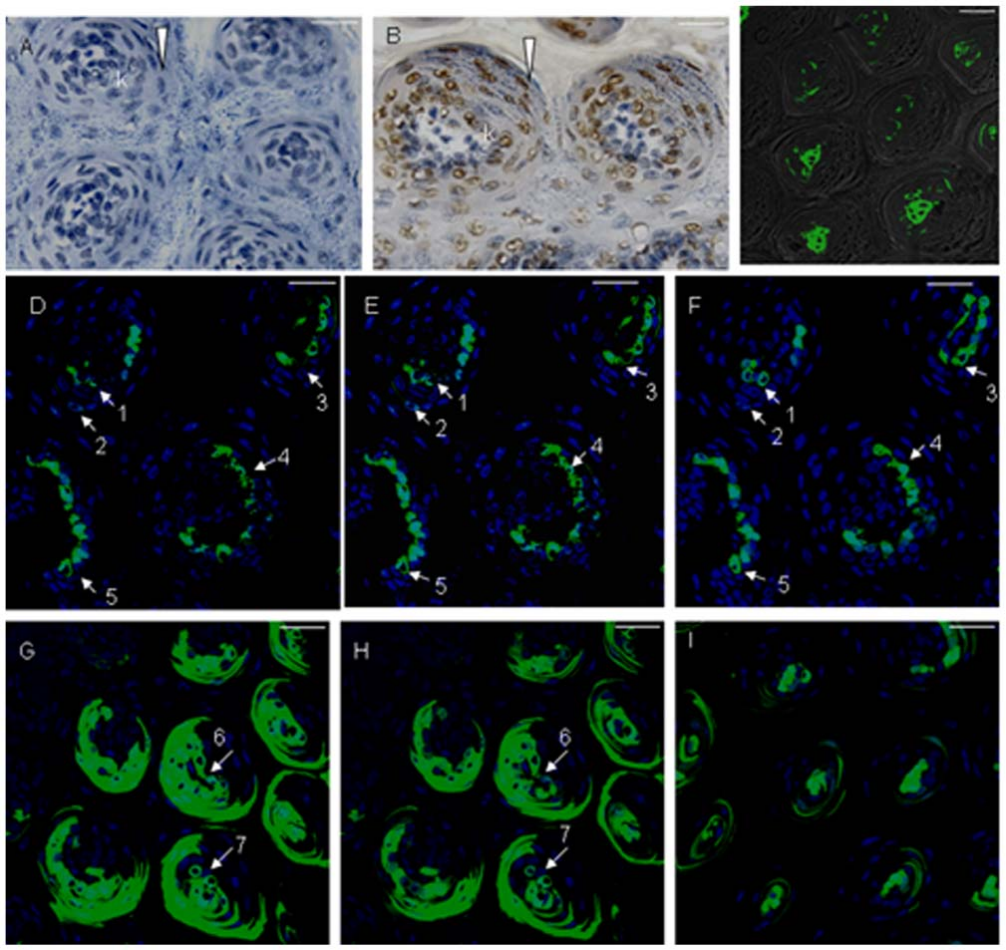
GFP was first observed in the interior and then the external of filiform papillae. In control mice, AcH4 was undetectable in filiform papillae (A). After 24 h of TGF-β signaling disruption, a higher acetylation level of histone H4 was observed in filiform papillae and interpapillary epithelia. Note that the cell migration stream was observed in filiform papillae (B, triangle). However, K14 expression was detected in connective tissue and filiform papillae in control mice (C). Serial confocal pictures first revealed GFP expression in keratinizing-like cells with a hollow and flexible fish-net like appearance, located in the interior of filiform papillae (D, E and F). (1–5) GFP+ cells with a hollow and flexible fish-net like appearance. GFP expression was later observed in the external of filiform papillae after 3 days (G and H) or 35 days (I) of Dox induction. However, only GFP+ cells with a fish-net like appearance in filiform papillae were observed after 3 days of Dox induction (G and H). (6–7) GFP+ cells with a hollow and flexible fish-net like appearance. Scale bar, 12 µm for A and B, 50 µm for C–I.

Stem cells do not migrate tangentially as they mature and must have integrated positional information at some point during development. This positional information becomes encoded in the progenitors [33]. In addition, epigenetic repression and derepression are important in controlling the balance between epidermal stem/progenitor cell proliferation and differentiation [34], [35]. In order to understand the maturation process of lingual epithelial cells, serial sections were labeled with epigenetic molecular markers (acetylated histone H4). Here, we focused only on the filiform papillae. In control mice, acetylated histone H4 (AcH4) was undetectable in filiform papillae (Figure 9A). After 24 h of TGF-β signaling disruption, a higher acetylation level of histone H4 was observed in filiform papillae and epithelia. The cell migration stream was also observed in filiform papillae (Figure 9B). Meanwhile, the number of keratinizing-like cells (35.1%, 394/1123) significantly increased after 24 h of TGF-β signaling disruption, compared with control mice (5.17%, 56/1082). It was noted that K14 expression was detected in the connective tissue and filiform papillae (Figure 9C). In serial confocal images, GFP was first observed in keratinizing-like cells with a hollow and flexible fish-net like appearance after 24 h of Dox induction, which were located at the interior of filiform papillae (Figure 9D–9F). GFP expression was later observed in the external of filiform papillae after 3 days (Figure 9G and 9H) and 35 days (Figure 9I) of Dox induction. However, we only observed GFP+ cells with a fish-net like appearance in filiform papillae after 3 days of Dox induction (Figure 9G and 9H).

**Figure 10:**
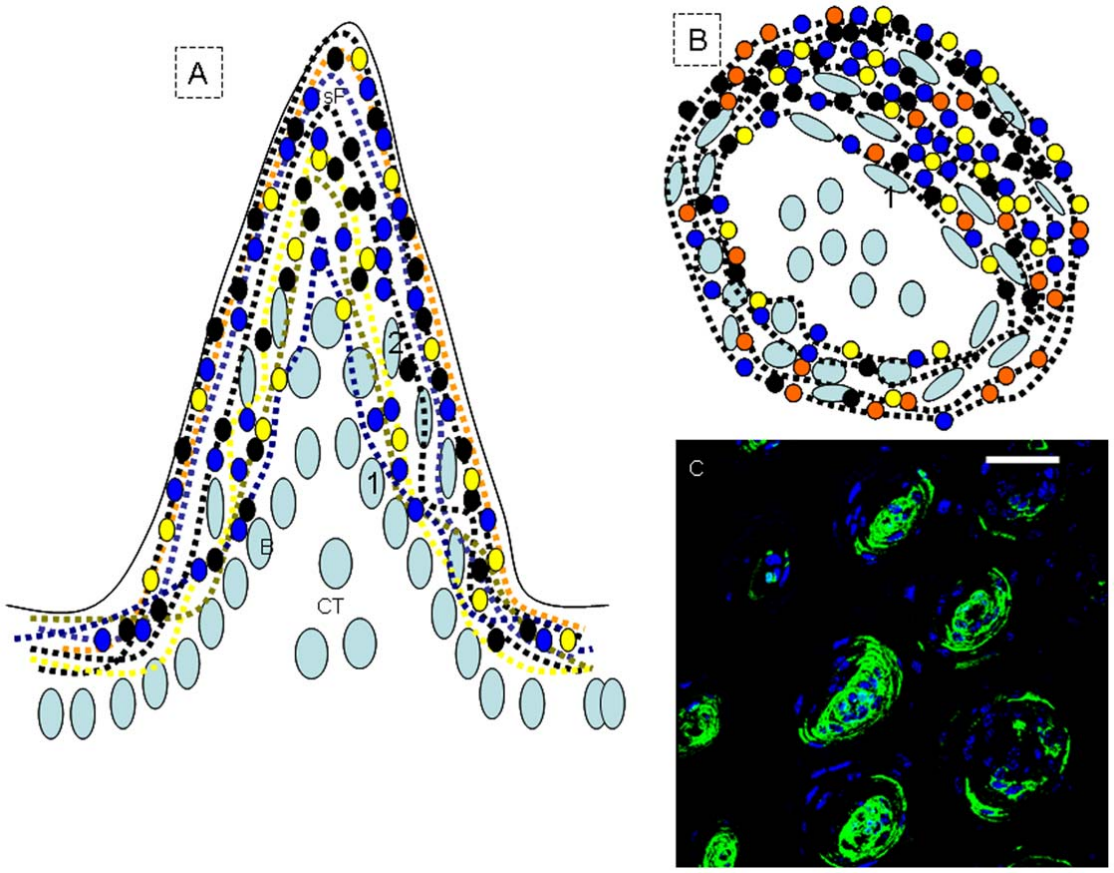
Cell migration stream of filiform papillae. Many stem cell populations formed the unique structure of filiform papillae. K14+ cells were fated to develop into interior cells, and then ectopic expression of K14 was observed in external cells. (A) Frontal section of filiform papillae. (B) Horizontal section of filiform papillae. After TGF-β signaling dirsruption for 24 h, GFP+ cells first appeared on the interior of filiform papillae, site 1; after 3 days, GFP+ cells appeared on the external of filiform papillae, site 2, and after 35 days, confocal analysis with anti-GFP showed the specific involvement of K14+ lineage cells in the formation of the special structure in filiform papillae (C). sF, spine of filiform papillae. Scale bar, 50 µm.

The current results together indicated that there are many stem/progenitor cell populations which form the unique structure of filiform papillae. K14+ progenitor cells are fated to develop into interior cells, and indeed ectopic expression of K14 was observed in the external cells (Figure 10A and 10B). Confocal analysis with anti-GFP showed the specific involvement of K14+ lineage cells in the formation of the unique structure in filiform papillae (Figure 10C).

## Discussion

In essence, the above results collectively showed that there is a cell migration stream in lingual epithelia, consisting of multiple stem cell pools and differentiation cell pools, which form a unique structure in filiform papillae. The cell migration stream in filiform papillae is related to that in epithelia. Disruption of TGF-β signaling in K14+ cells exhausts the downstream cell pools (below the K14 cell pool), resulting in proliferation of upstream cell pools due to the requirement of maintaining a dynamic balance among the cell pools in tongue epithelia (Figure S5). Such dynamics among the epithelial cell pools have been described in adult olfactory neuroepithelium [36] and bulge epithelial stem cells [37], [38]. In adult olfactory neuroepithelium, horizontal basal cells (HBCs) remain largely quiescent during replenishment of olfactory receptor neurons (ORNs) associated with normal turnover or after the acute, selective loss of mature neurons. Neuronal repopulation depends on proliferation of globose basal cells (GBCs), which is sufficient to support the requirement for replacement. In skin epithelium, a progenitor population residing in the interfollicular epidermis is sufficient to sustain its renewal. After epidermal injury, however, a distinct population residing in hair follicles, the normally quiescent bulge epithelial stem cells, is activated to proliferate, and its progeny directly contribute to repopulation of the epidermis.

According to the model of stem cell regulation [3], [6], there are two strategies for stem cell self-renewal, asymmetric cell division and population asymmetry. K14+ stem cell division results in two daughter cells having unequal fates; one cell remains in the stem cell compartment, whereas the other commits to the fate of keratinocytes. Thus, it was reported that K14+ stem cells can continually generate keratinocytes and TB cells [8]. In the current study, we disrupted the TGF-β signaling pathway in K14+ progenitor cells, which has been reported to play a specific role in coordinating the development of epithelial cells [39]. Interruption of TGF-β signaling therefore should change the strategy of K14+ progenitor cell division and commit the daughter cells to the fate of becoming keratinocytes. Therefore, within a short period of time, the Dox treatment should have quickly exhausted the K14+ progenitor cell pools. Meanwhile, population asymmetry would have allowed nearby cells to replenish the K14+ progenitor cell pools (Figure S5). However, the internal regulation of many cells was apparently altered in the lingual epithelia as revealed by the epigenetic marker, indicating a changed microenvironment (unpublished data, FL). The consequence of the changed microenviroment was ectopic expression of the *K14* gene, as we observed GFP expression in the filiform papillae after 3 days of Dox induction. In CV papillae, the appropriate cues may not have been present to induce the migrating cells to express appropriate markers there at the proper time. Previous studies have suggested that different epithelia stem cell populations are functionally equivalent and interconvertible, and their differentiation potential is largely determined by their local microenvironment [31]. An epithelial cell that has not lost the ability to divide and embarked on a program of terminal differentiation can self-renew and exhibit other stem cell properties. If this is the case, then the markers of the different stem cell pools may be expressed in response to the local environment rather than being inherent characteristics [32], [40]. It has been noted that thymic epithelial stem cells can function as epidermal stem cells as well as multipotent hair follicle stem cells when exposed to an inductive skin microenvironment [7].

It seems rather unique that *K14* showed a different expression pattern from tip to posterior along the tongue. K14+ lineage cells contributed to development of papillae and epithelia, as well as to TB cells. The results of the study collectively demonstrated that the expression of *K14* gene was variable, depending on the location on the tongue and papillae after disruption of TGF-β signaling. This phenomenon may not be restricted to the lingual epithelia. After prolonged induction of ErbB2 (an epidermal growth factor receptor) in K14+ cells of adult mice, severe hyperplasias in the stratified epithelia have been observed and, notably, expression levels of several genes such as *K14, K10, K16* and *filaggrin* have been shown to drastically change [20]. In another study, after 21 days of inducing KLF4 (Krueppel-like factor 4) in K14+ cells of adult mice, immunohistological analysis revealed drastic changes in the expression levels of *K14, K1, K16* and *K17* genes and the proliferation marker PCNA (Proliferating Cell Nuclear Antigen) in the ventral skin [41]. Furthermore, BrdU incorporation has been shown to significantly increase within the epithelia of both the anal canal and anal skin of 7-week-old K14-Cre/TβRII (fl/fl) conditional knockout (cKO) mice when compared to their wild-type (WT) littermates. In addition, elevated β1-integrin-FAK-Src-MAPK activity was also observed in backskin epithelium in this cKO mice [42]. Recently, it was reported [7] that thymic epithelial cells (TECs) form a complex three-dimensional network organized in cortical and medullary compartments, the organization of which is notably different from simple or stratified epithelia. However, TECs which maintain a K5/K14+ profile in serial cultivation *in vitro* can be integrated into a thymic epithelia network again and adopt the fate of hair follicle multipotent stem cells when exposed to an inducing skin microenvironment.

In conclusion, this study showed that there is a cell migration stream in lingual epithelia, consisting of multiple stem cell pools and differentiation cell pools, which forms a unique structure in filiform papillae. The cell migration stream in filiform papillae is related to that in epithelia. *K14* is differentially expressed from the tip to posterior along the tongue, and K14+ lineage cells contribute to papillae and epithelia, indicating that the variable expression of *K14* gene is dependent on the location in the tongue and papillae. Thus, the local microenvironment provides important cues for cell differentiation in lingual epithelia. Undoubtedly, a systematic interpretation of the relationship between gene expression profile and development of lingual epithelial stem cells *in vivo* will further help us to understand basic questions of developmental biology and molecular mechanisms in support of tongue diagnosis.

## Methods

### Double transgenic mice and Dox treatment

K14-rtTA and TetO-EGFP-Tgfbr2 (PTR) mouse lines were obtained from Jackson Laboratories (Bar Harbor, Maine). The transgenic mice were bred and maintained at the Monell Chemical Senses Center animal facility. All procedures involving animals were approved by the Monell Chemical Senses Center Institutional Animal Care and Use Committee.

Five to seven-month-old mice were used in the current study. Each time point involved 3–4 male mice. Female mice were evaluated at the following time points: 2 days (n = 3), 15–18 days (n = 3), 30–35 days (n = 3). For Dox administration (Sigma, St. Louis, MO, USA), the drug was diluted in 5% sucrose in water to a final concentration of 0.3–0.5 mg/ml and supplied as drinking water. Animals were allowed unlimited access to the Dox-containing water, which was changed every 2–3 days. A single intraperitoneal injection (10 mg/kg body weight) was also given within 3 days after administration of Dox in the drinking water.

### Histology, immunostaining and BrdU labeling procedure

For immunocytochemistry, mice were perfused transcardially with 2–4% paraformaldehyde (PFA) in phosphate-buffered saline (PBS; pH 7.2–7.4). The tongue tissues were dissected, post-fixed in PFA for 2–12 h and cryoprotected in 30% sucrose in PBS at 4°C overnight. After sectioning on a cryostat, 10–12 µm sections were collected onto Superfrost Plus Microscope slides (Fisher Scientific). Polyclonal primary antibodies used were specific for GFP (goat, Abcam, Cambridge, MA, USA ab-5450; rabbit Abcam ab-6556), anti-acetyl histone H4 (rabbit, Millipore, Bedford, MA, USA, 06-866). Monoclonal primary antibodies used were against BrdU (Sigma B2531). Staining was performed with the TSA Plus system from Perkin Elmer according to the manufacturer's instructions. Fluorescent images were captured with the Leica TCS SP2 Spectral Confocal Microscope (Leica Microsystems Inc., Mannheim, Germany).

Stainings for GFP and anti-acetyl histone H4 were performed with the standard immunocytochemical procedure using VECTASTAIN Elite ABC Kits (Burlingame, CA, USA) according to the manufacturer's instructions. For BrdU labeling, mice were injected intraperitoneally with 50 mg per kg of body weight of BrdU (Sigma) and sacrificed 2–3 h later for testing.

Standard hematoxylin and eosin staining was performed in the current study. Brightfield images of the sections were digitally captured. Percent of keratinizing-like nuclei/total nuclei was quantitatively calculated with ImagePro Plus (Media Cybernetics Inc., Silver Spring, MD, USA) in serial sections of filiform papillae in posterior of tongue. Data are presented as the mean ± SEM. Data were analyzed by one way analysis of variance using SPSS11.0 software. Differences were considered to be significant when *P*<0.05.

## Supporting Information

Figure S1 **Tet-On inducible transgenic mouse system.** A schematic illustration of the transgenes in K14- rtTA-PTR mice. The PTR transgene contains the bidirectional tetO promoter (pBi), which drives a dominant-negative inhibitor of the type II TGFb receptor (*ΔTgfbr2*) and enhanced green fluorescent protein (*EGFP*) genes. The binding of rtTA to the tetracycline responsive element (TetRE) and the induction of the *EGFP/ΔTgfbr2* transgene should only occur in the presence of Dox. **TGF-β signaling pathway and Tgfbr2 dominant-negative receptor**. The three TGF-β Isoforms use a common receptor. The receptors are divided into two types: type I and type II receptors. Type I and II receptors contain three domains: extracelluar domain, intracellular domain and kinase domain. First, TGF-β interacts with the type II receptor. The type II receptor activates the type I receptor, which in turn activates the downstream signaling pathway (Smad-dependent pathway and MAPK pathway). The dominant-negative receptor for Tgfbr2 is mutated in the intracellular domain of the type II receptor. While the mutated receptor can normally bind with TGF-β, it cannot activate the type I receptor. Moreover, binding of TGF-β with the mutated type II receptor is irreversible, thus blocking the downstream TGF-β signaling pathway.
(TIF)

Figure S2 **GFP expression is detected in the spine of filiform papillae after 7 days of Dox induction.** After 24 h of TGF-β signaling disruption, GFP expression was detected in the basal cell layer, base of filiform papillae and TB of fungiform papillae (arrow). After 7 days of TGF-β signaling disruption, GFP expression was detected in the basal cell layer and base of filiform papillae. Furthermore, GFP expression was also detected in the spine of filiform papillae. Fu, fungiform papillae. F, filiform papillae. bF, base of filiform papillae. sF, spine of filiform papillae. eFu, epithelia of fungiform papillae.
(TIF)

Figure S3 **Chimeric GFP expression in female mice tongue after 18 days of Dox induction.** Immunohistochemistry with anti-GFP showed the distribution of K14+ lineage cells in the basal cell layer, filiform papillae and spine of filiform papillae (A and B). B, basal cell layer. CT, connective tissue. F, filiform papillae. TB, taste bud. sF, spine of filiform papillae. Scale bar, 12 µm.
(TIF)

Figure S4 **Formation of a cell migration stream in lingual epithelia and filiform papillae by BrdU+ proliferating cells after 35 days of Dox induction.** (A) In control mice, 2H BrdU only labeled proliferating cells in connective tissue. (B) After 35 days of TGF-β signaling disruption, a cell migration stream was formed by 2H BrdU labeled proliferating cells. F, filiform papillae. Ie, interpapillary epithelia. CT, connective tissue. Scale bar, 12 µm.
(TIF)

Figure S5 In K14-rtTA-PTR transgenic mice, K14-rtTA is X-linked gene. In male mice, 5 h of TGF-β signaling disruption induced the proliferation of inherent K14+ cells, andthose cells developed into the basal cell layer 24 h later (A). Two to three days of TGF-β signaling disruption exhausted K14+ progenitor cell pools, which induced the proliferation of upstream or adjacent stem cell pool. Meanwhile, the internal regulation of many cells was apparently altered in the lingual epithelia as revealed by an epigenetic marker, indicating an altered microenviroment, the consequence of which was ectopic expression of the *K14* gene and GFP (B). In female mice, half of the K14+ progenitor cell pool remained due to X-inactivation after disruption of TGF-β signaling (C and D).
(TIF)
