# Supplementary figures and images for: Local Microenvironment Provides Important Cues for Cell Differentiation in Lingual Epithelia
Source: PLoS One. 2012 Apr 13;7(4):e35362. doi: 10.1371/journal.pone.0035362 (PMC3325925; doi:10.1371/journal.pone.0035362)

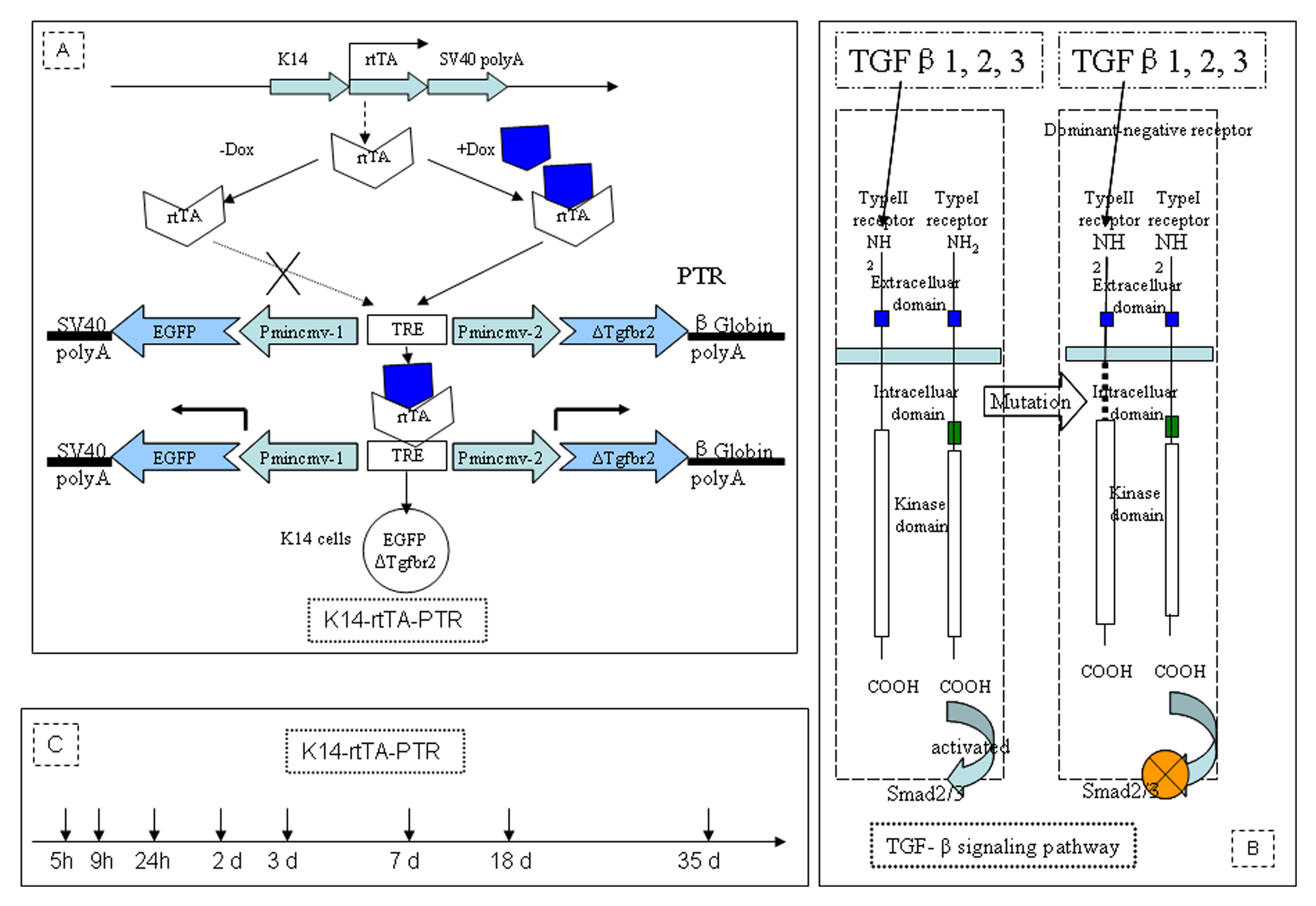

Supplement: Figure S1 — Tet-On inducible transgenic mouse system. A schematic illustration of the transgenes in K14- rtTA-PTR mice. The PTR transgene contains the bidirectional tetO promoter (pBi), which drives a dominant-negative inhibitor of the type II TGFb receptor (ΔTgfbr2) and enhanced green fluorescent protein (EGFP) genes. The binding of rtTA to the tetracycline responsive element (TetRE) and the induction of the EGFP/ΔTgfbr2 transgene should only occur in the presence of Dox. TGF-β signaling pathway and Tgfbr2 dominant-negative receptor. The three TGF-β Isoforms use a common receptor. The receptors are divided into two types: type I and type II receptors. Type I and II receptors contain three domains: extracelluar domain, intracellular domain and kinase domain. First, TGF-β interacts with the type II receptor. The type II receptor activates the type I receptor, which in turn activates the downstream signaling pathway (Smad-dependent pathway and MAPK pathway). The dominant-negative receptor for Tgfbr2 is mutated in the intracellular domain of the type II receptor. While the mutated receptor can normally bind with TGF-β, it cannot activate the type I receptor. Moreover, binding of TGF-β with the mutated type II receptor is irreversible, thus blocking the downstream TGF-β signaling pathway. (TIF) [file pone.0035362.s001.tif]

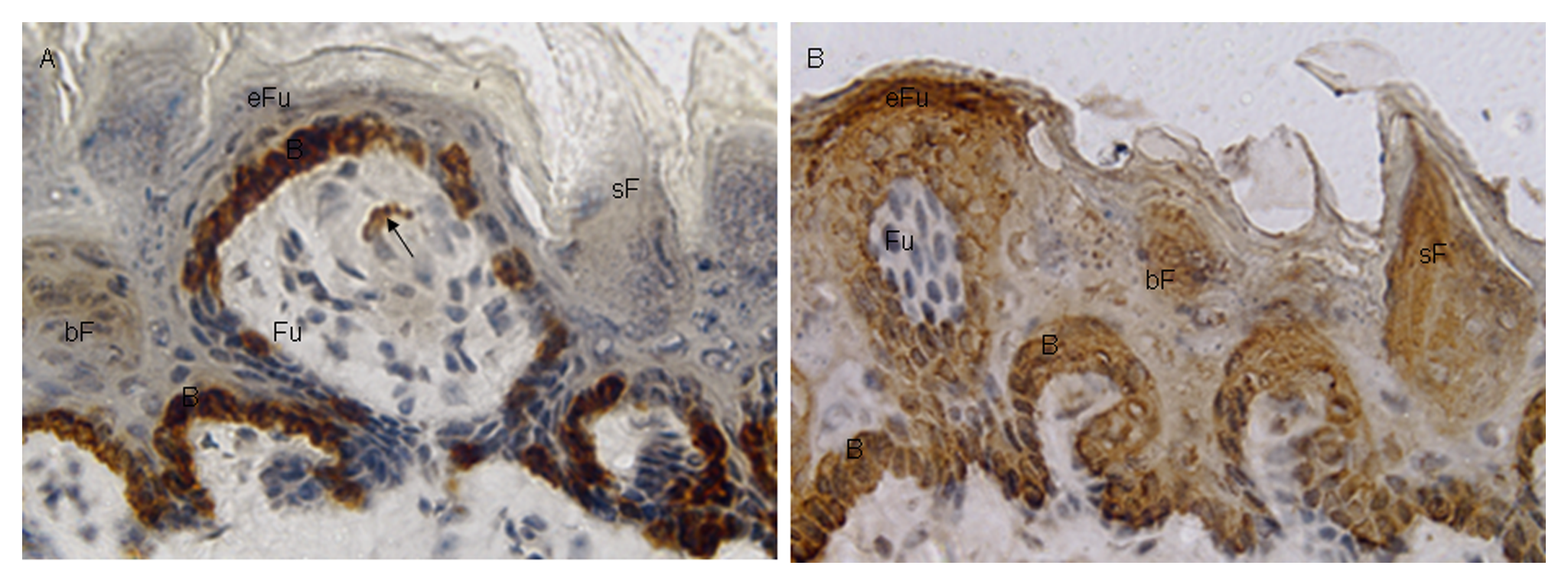

Supplement: Figure S2 — GFP expression is detected in the spine of filiform papillae after 7 days of Dox induction. After 24 h of TGF-β signaling disruption, GFP expression was detected in the basal cell layer, base of filiform papillae and TB of fungiform papillae (arrow). After 7 days of TGF-β signaling disruption, GFP expression was detected in the basal cell layer and base of filiform papillae. Furthermore, GFP expression was also detected in the spine of filiform papillae. Fu, fungiform papillae. F, filiform papillae. bF, base of filiform papillae. sF, spine of filiform papillae. eFu, epithelia of fungiform papillae. (TIF) [file pone.0035362.s002.tif]

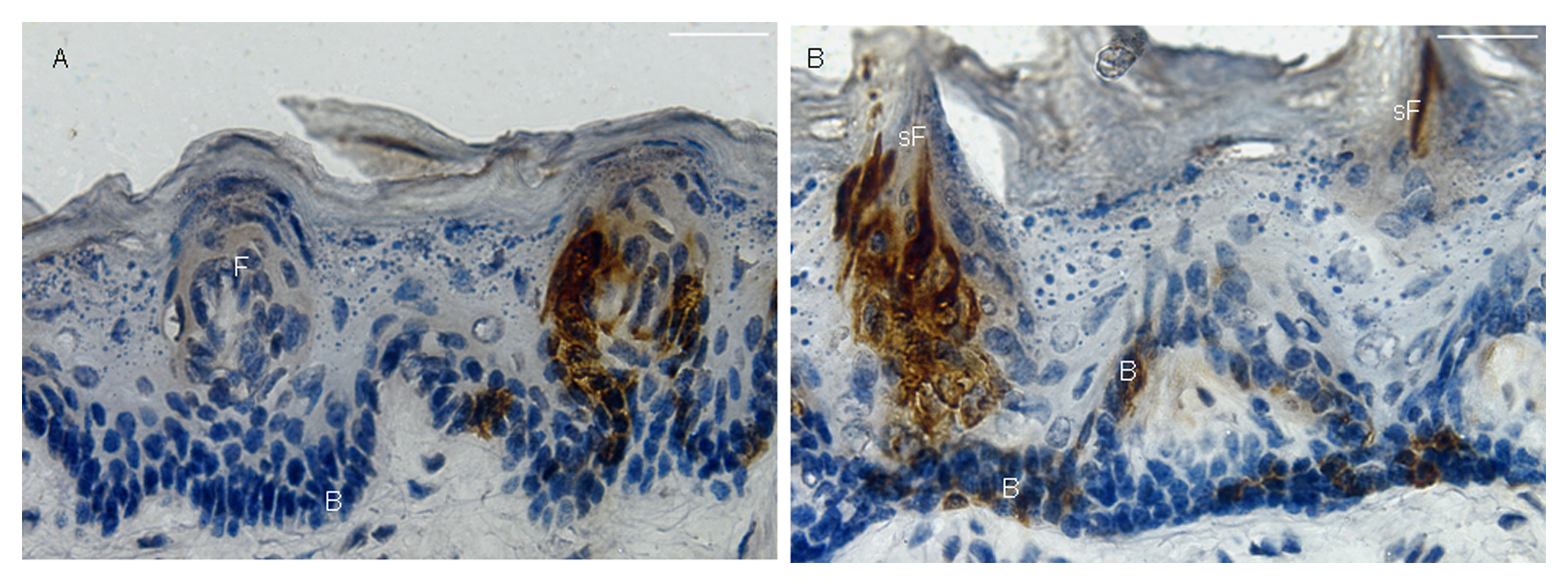

Supplement: Figure S3 — Chimeric GFP expression in female mice tongue after 18 days of Dox induction. Immunohistochemistry with anti-GFP showed the distribution of K14+ lineage cells in the basal cell layer, filiform papillae and spine of filiform papillae (A and B). B, basal cell layer. CT, connective tissue. F, filiform papillae. TB, taste bud. sF, spine of filiform papillae. Scale bar, 12 µm. (TIF) [file pone.0035362.s003.tif]

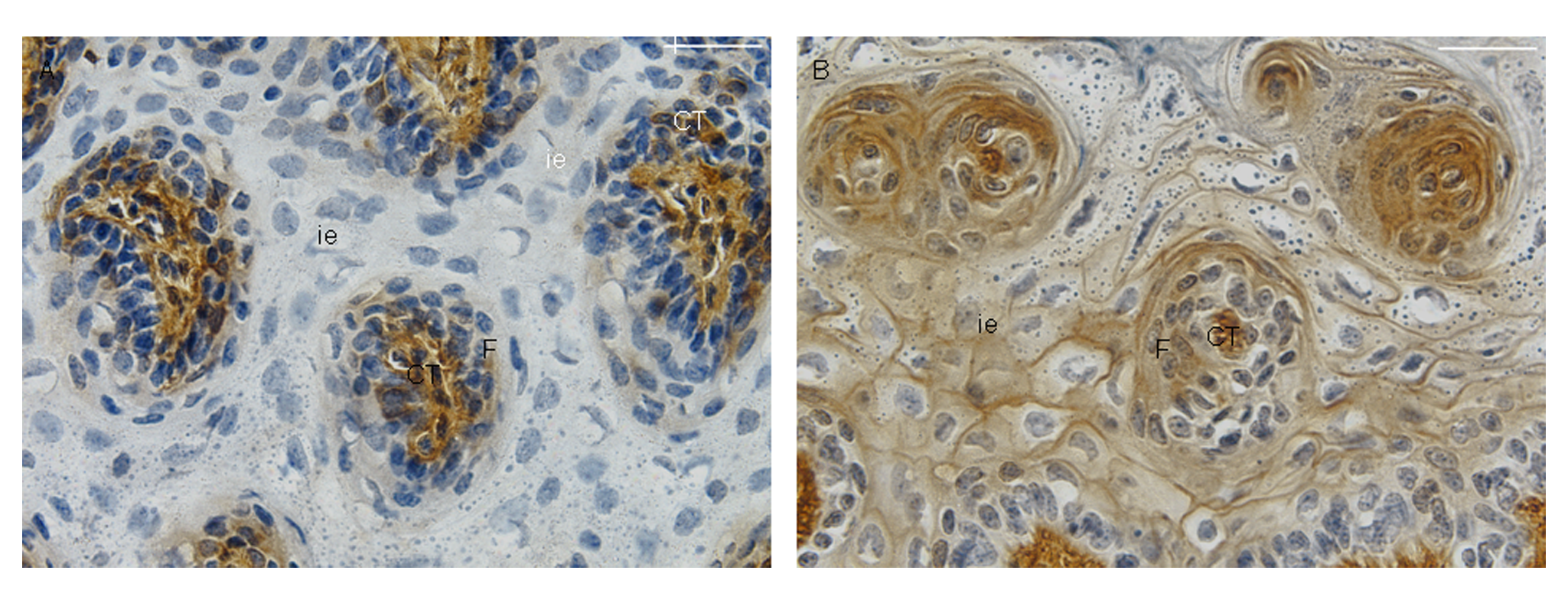

Supplement: Figure S4 — Formation of a cell migration stream in lingual epithelia and filiform papillae by BrdU+ proliferating cells after 35 days of Dox induction. (A) In control mice, 2H BrdU only labeled proliferating cells in connective tissue. (B) After 35 days of TGF-β signaling disruption, a cell migration stream was formed by 2H BrdU labeled proliferating cells. F, filiform papillae. Ie, interpapillary epithelia. CT, connective tissue. Scale bar, 12 µm. (TIF) [file pone.0035362.s004.tif]

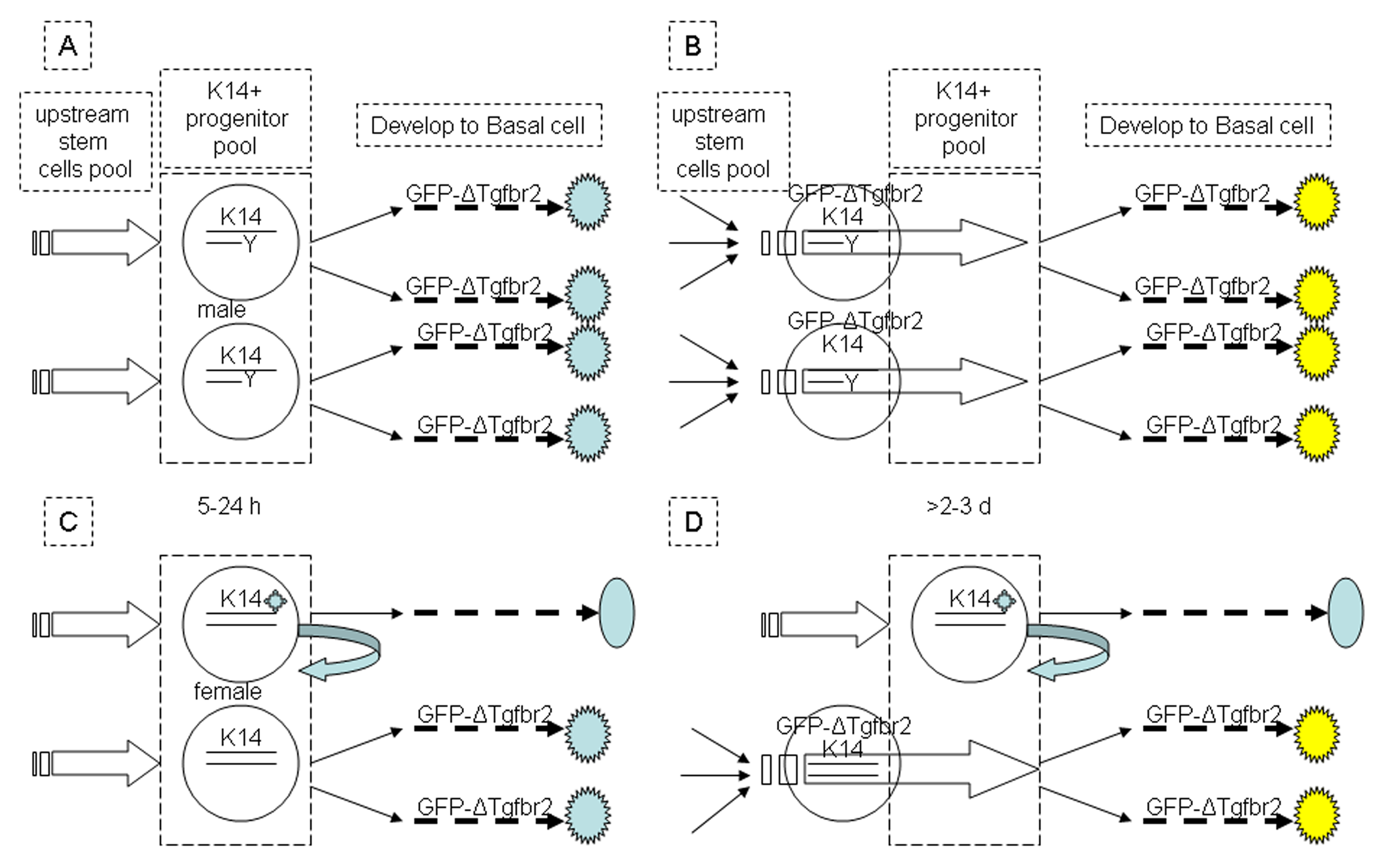

Supplement: Figure S5 — In K14-rtTA-PTR transgenic mice, K14-rtTA is X-linked gene. In male mice, 5 h of TGF-β signaling disruption induced the proliferation of inherent K14+ cells, andthose cells developed into the basal cell layer 24 h later (A). Two to three days of TGF-β signaling disruption exhausted K14+ progenitor cell pools, which induced the proliferation of upstream or adjacent stem cell pool. Meanwhile, the internal regulation of many cells was apparently altered in the lingual epithelia as revealed by an epigenetic marker, indicating an altered microenviroment, the consequence of which was ectopic expression of the K14 gene and GFP (B). In female mice, half of the K14+ progenitor cell pool remained due to X-inactivation after disruption of TGF-β signaling (C and D). (TIF) [file pone.0035362.s005.tif]
